# Inferring the evolutionary history of *Populus trichocarpa* from whole genome resequencing data

**DOI:** 10.1186/1753-6561-5-S7-O1

**Published:** 2011-09-13

**Authors:** Stephen DiFazio, Gancho Slavov, Eli Rodgers-Melnick, Joel Martin, Wendy Schackwitz, Ranjan Priya, Gerald Tuskan

**Affiliations:** 1 Department of Biology, West Viriginia University, USA; 2 Joint Genome Institute, USA; 3 Oak Ridge National Lab, USA

## 

The signatures of evolutionary change are encoded in the structure and composition of the *Populus trichocarpa* genome, which is currently the most extensively characterized among forest trees. We report on genome-scale analyses of 580 trees collected from across the range of this species, including whole genome resequencing of over 50 trees. Several lines of evidence suggest a relatively strong bottleneck in the recent evolutionary past of this species, including reduced nucleotide diversity compared to other *Populus* species and relatively high Linkage Disequilibrium (LD). Furthermore, there is a preponderance of strong latitudinal gradients in allele frequencies, and loci with strong gradients tend to have high frequencies of derived alleles and occur in regions of high LD. This suggests a complex interplay between post-glacial migration and selection in shaping nucleotide diversity in this species.

Further insights can be gained by examining prevailing patterns within the genome of this species. For example, the recent whole genome duplication is well-documented, but close examination of patterns of loss and retention of duplicated genes lends support to the Gene Balance Hypothesis as a driving force behind the maintenance of duplicated gene pairs, with positive selection driven by subfunctionalization playing a secondary role. Retained duplicate pairs tend to belong to functional classes that are characterized by high protein-protein interactions, as predicted by Gene Balance (members of signal transduction cascades and transcription factors). Patterns of expression across a diverse set of tissues are consistent with two classes of duplicated genes: one group with retained ancestral functions, and a smaller group with divergent functions driven by alternate degeneration of ancestral functions.

A major factor reflecting genome structure and content is the population recombination rate. We will show that this rate is highly variable across the genome, characterized by large regions of relatively low recombination punctuated by hotspots with greatly elevated recombination. There is a strong relationship between recombination rates determined by a very dense genetic map and recombination inferred from population resequencing data. Recombination rate is inversely correlated with methylation status and repeat density, with the correlation being strongest for LTR Gypsy elements and AT low complexity repeats. Hotspots of recombination appear to be associated with a short sequence motif, and preferentially occur 5’ of genes.

Whole genome resequencing is steadily increasing the resolution with which we can dissect the evolutionary legacy of this important species, and is therefore enhancing our understanding of current distributions and, potentially, future responses to artificial and natural selection. As information continues to accumulate about this and other related species, including congeners and a host of organisms with ecological associations, we will begin to piece together the complex web of genetic, ecological, and evolutionary forces that shape extant communities. We will thereby approach the ultimate goal of understanding the molecular bases of adaptive evolutionary change in a community context.

